# Lateral Femoral Cutaneous Nerve Block for Postoperative Pain Control After Total Hip Arthroplasty Using the Direct Anterior Approach: A Single-blinded Randomized Control Trial

**DOI:** 10.14789/jmj.JMJ22-0033-OA

**Published:** 2023-04-14

**Authors:** SONOKO SAKURABA, TAKESHI OMAE, SHUKO NOJIRI, KEITO KOH, SHO YAMAZAKI, MASATERU KUMEMURA

**Affiliations:** 1 Department of Anesthesiology and Pain Clinic Juntendo University Shizuoka Hospital, Izunokuni, Shizuoka, Japan; 1 Department of Anesthesiology and Pain Clinic Juntendo University Shizuoka Hospital, Izunokuni, Shizuoka, Japan

**Keywords:** lateral femoral cutaneous nerve, nerve block, lateral femoral nerve, total hip arthroplasty, direct anterior approach

## Abstract

**Background:**

Total hip arthroplasty (THA) employing the direct anterior approach (DAA) is increasingly performed as a less invasive procedure with faster recovery than other approaches. Unlike other approaches, the skin incision is made on the lateral thigh, distal to the inguinal ligament. However, the effectiveness of ultrasound-guided lateral femoral cutaneous nerve (LFCN) block for postoperative analgesia after THA using DAA has not been investigated.

We hypothesized that ultrasound-guided LFCN block using DAA would reduce postoperative pain after THA.

**Methods:**

A prospective, randomized, observer-blinded controlled trial was conducted. The 92 patients included were divided into two groups: those who received only femoral nerve block (FNB group) and those who received femoral nerve block and LFCN block with 10mL of 0.25% levobupivacaine (FNB + LFCNB group). Both groups received intravenous patient-controlled analgesia (fentanyl) postoperatively. A numerical rating scale was used to quantify pain at 3 and 48 h postoperatively.

**Results:**

There was no significant difference in pain at rest and during movement between the FNB and FNB + LFCNB groups (at rest: Z = -1.6814, p=0.0927; during on movement: Z = -0.9677, p=0.9487). There was also no significant difference in pain severity at rest and during movement between the FNB and FNB + LFCNB groups postoperatively.

**Conclusions:**

LFCNB did not improve postoperative pain relief in patients undergoing THA with DAA.

## Introduction

Pain control after total hip arthroplasty (THA) has previously been managed with epidural anesthesia. However, introduction of early postoperative anticoagulant therapy has led to the avoidance of catheter insertion into the central nervous system. Pain relief is now provided by various methods such as periarticular injections and intravenous patient-controlled analgesia (IV-PCA) with opioids. Peripheral nerve blocks are also an option^1)^. Peripheral nerve blocks have been shown to reduce pain following THA and have effects comparable to those of anesthetic delivery to the central nervous system^2)^.

Compared with other approaches, THA via the direct anterior approach (DAA) is known to have a faster recovery time, with earlier ambulation and shorter hospital stay^3, 4)^. Consequently, it has been adopted as a less invasive option in an increasing number of cases over the past decade^5, 6)^.

**Figure 1:**
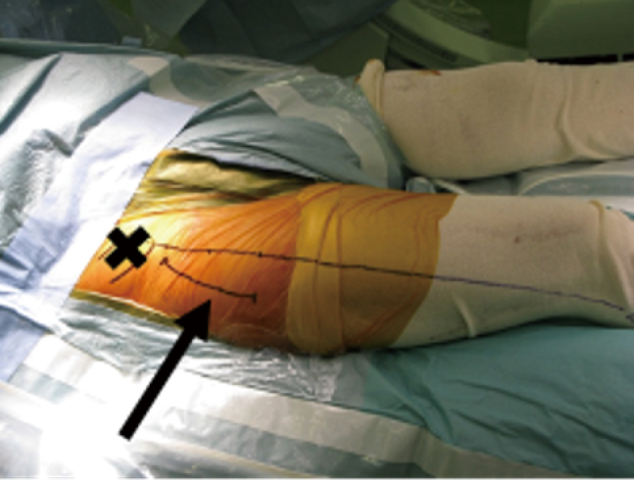
Total hip arthroplasty by direct anterior approach incision Incision line is indicated by the black arrow. Anterior superior iliac spine is indicated by a black X.

In the posterior and lateral approaches, the incision is made on the back of the hip. With the DAA, the incision site is located on the lateral aspect of the thigh^5)^. Figure 1 shows the skin incision site in a patient who underwent THA using DAA.

Pain sensation on the outer thigh is supplied by the lateral femoral cutaneous nerve (LFCN). Due to the great anatomical variability of the LCFN, the success rate for conventional LFCN blocks was historically low^7)^; however, it has dramatically increased when used with ultrasound guidance^8)^.

Currently, LFCN blocks are commonly employed for postoperative analgesia. LFCN blocks have been found to decrease postoperative analgesic use in surgeries for femoral neck fractures wherein the skin incision is on the lateral thigh, similar to THA with DAA^9)^. Although the efficacy of LFCN blocks for pain management after THA has been investigated several times^10, 11)^, THA in these studies was performed with either the posterior or unspecified approaches; none of them used DAA.

In this study, we hypothesized that LFCN blocks for THA using DAA reduce postoperative pain. We compared the numerical rating scale (NRS) scores 3 h postoperatively between groups that did and did not receive an LFCN block.

## Materials and Methods

### Ethics approval and consent to participate

This study was approved by the hospital Ethics Committee and prospectively registered with the University Hospital Medical Information Network (UMIN: UMIN000035562) at its initiation. This study was conducted in accordance with the guidelines of the Declaration of Helsinki (2013). All study participants provided written informed consent for their participation in the study.

### Study design and participants

From December 10, 2018, to August 9, 2019, we conducted a prospective, observer-blinded, comparative, and randomized controlled trial using stratified randomization at the Department of Anesthesiology and Pain Clinic, Juntendo Shizuoka Hospital, Shizuoka, Japan. The exclusion criteria were as follows: (1) previous THA on the same side; (2) presence of a lesion not suitable for skin puncture around the injection site, as determined by a surgeon; (3) history of allergic reactions to local anesthetics; (4) under anesthesia for more than 6 h; (5) dementia (Hasegawa Dementia Scale-Revised score ≤ 22 points); (6) requiring postoperative mechanical ventilation; (7) prothrombin time-international normalized ratio ≥ 2.0, activated partial thromboplastin time ≥ 1.5 × the reference value, platelet count ≤ 50,000/μL, and other clear indications for the presence of a coagulation disorder; and (8) evaluated to be ineligible by the principal investigators for any other reason. Stratified randomization was performed according to age and sex, which were considered to influence postoperative pain. The participants were patients scheduled to undergo THA using DAA at our hospital.

### Patient randomization

Preoperatively, patients were randomly assigned to receive a femoral nerve block (FNB group) or FNB plus LFCN block (FNB + LFCNB group). Stratified randomization was performed using a 1:1 allocation table according to sex, and age. The patients were distributed according to the allocation table by investigators who were not directly involved in their care. The analysis was performed using an intention-to-treat analysis.

### Application of anesthesia

Upon entering the operating room, all patients were connected to general monitoring equipment. They received general anesthesia with propofol, fentanyl, remifentanil, and rocuronium, and then underwent tracheal intubation.

Preoperatively, the FNB group received FNB alone, and the FNB + LFCNB group received FNB and LFCNB. Intraoperatively, anesthesia was maintained with sevoflurane, desflurane, or propofol-rocuronium-remifentanil-fentanyl. The dose of fentanyl used during surgery was at the discretion of the anesthesiologist.

### Block interventions

Four experienced anesthesiologists performed the nerve blocks. For ultrasound guidance, a 6-15-MHz linear probe of SonoSite S-Nerve (FUJIFILM Sonosite, Inc., Bothell, WA, USA) or a 4.2–13.0-MHz linear probe of the LOGIQ e premium (GE Healthcare Co., Chicago, IL, USA) was used. For the injection, a 20 G × 80 mm UNIEVER nerve block needle (Unisys Corp., Tokyo, Japan) was used.

**Figure 2:**
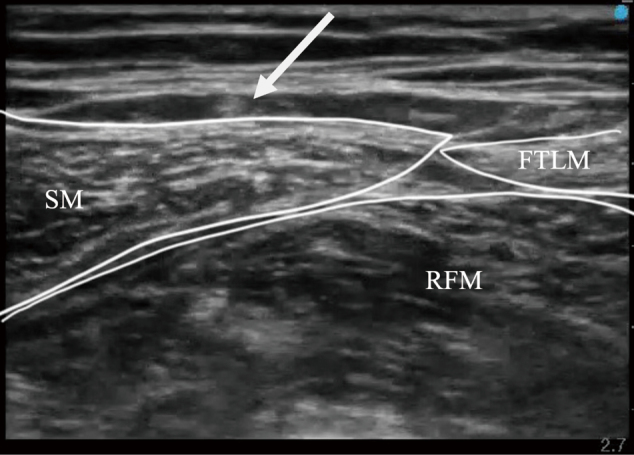
LFCN in FFFT ← : lateral femoral cutaneous nerve SM : sartorius muscle, FTML : tensor fasciae latae muscle, RFM : rectus femoris muscle

The LFCN was identified approximately 10 cm caudal to the inguinal ligament using the fat-filled flat tunnel (FFFT, the space surrounded by the sartorius, tensor fascia lata, and rectus femoris muscles, and formed by a double layer of the fascia lata between the sartorius and tensor fascia lata muscles). Figure 2 shows the LFCN within the FFFT and at the level of the injection during the LFCNB.

After identifying the LFCN in FFFT, the echo probe was moved cranially to a position from where the LFCN could be identified sonographically. The blockade needle was inserted in-plane to inject 10 mL 0.25% levobupivacaine into the area surrounding the nerve. If the upper portion of the area could not be visualized, the drug was injected at the most superior position visible on ultrasonography, approximately 2 cm caudal to the inguinal ligament.

### Evaluation of pain

After recovering from the effects of general anesthesia, the patients were transferred to the recovery room, where postoperative assessments were conducted by a blinded observer, who was either a scrub or ward nurse. Subsequently, NRS scores at rest and on movement were measured at 3, 6, 12, 24, and 48 h postoperatively.

### Postoperative analgesia

Postoperatively, all patients received fentanyl 20 μg/h with a bolus dose of 20 μg via IV-PCA for 48 h.

### Outcome assessment

Postoperative pain was quantified using the NRS. The primary outcome was defined as NRS scores at rest and on movement 3 h postoperatively, and the secondary outcome as changes in NRS scores up to 48 h postoperatively.

### Sample size calculation and statistical analysis

Sample size calculation was performed using R 4.0.3. The following parameters were derived from our experience and data from previous studies^12, 13)^: two-tailed α=0.05, β=0.20, mean standard deviation of NRS=2.0, and δ=1.5. Using the Mann-Whitney- Wilcoxon test, the calculated sample size comprised 82 patients (41 in each group). Considering a 30% dropout rate, the final sample size was 108 patients. Intermediate analyses was not conducted. The standard deviation (SD) and interquartile range (IQR) were calculated for the average and median, respectively. For all tests and estimations, the significance level was set at 5% and a 95% confidence interval was used. In the event of missing NRS values, the multiple completion method was used to complete the data. The Mann-Whitney- Wilcoxon test was used for continuous variables, whereas the chi-square test or Fisher's exact test was used for ordinal and nominal variables. The secondary endpoint was the change in NRS at 48 h postoperatively, which was tested nonparametrically using the U test for MW and the Bonferroni method for correction of multiple comparison tests.

All analyses (except sample size calculations) were performed using the SAS software (SAS Institute Inc, NC, USA).

## Results

**Figure 3:**
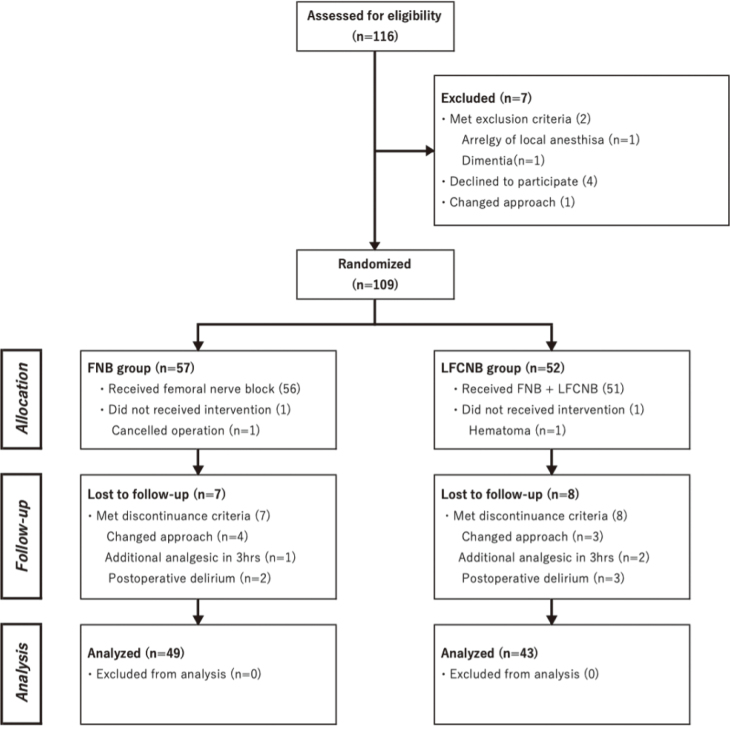
CONSORT diagram FNB, femoral nerve block; LFCNB, lateral femoral cutaneous nerve block

Of the 116 patients selected, 109 were enrolled in the study. When an additional sedative, not included in the protocol was used for a patient before the measurement of the primary outcome (NRS score 3 h postoperatively), the patient was excluded. Finally, 49 patients in the FNB group and 43 in the FNB + LFCNB group were included in the analysis (Figure 3).

There were no differences in patient demographics between the two groups (Table 1).

**Table 1:** Basic demographics

		FNB + LFCNB group (n=43)		FNB group (n=49)		p value
Age*		67.21	(10.75)	67.33	(10.53)	0.96
Sex						
	M	12		12		
	F	31		37		
Body weight (kg)*		60.2	(10.36)	57.86	(10.58)	0.29
Body height (cm)*		157.0	(0.083)	156.9	(0.080)	0.96
ASA						
	1	7		10		
	2	31		37		
	3	5		2		
Operation time (min)*		119.04	(24.75)	115.55	(19.23)	0.12
Duration from block to start of operation (min)*		46.66	(5.86)	45.30	(6.58)	0.41

* Mean (SD) ASA, American Society of Anesthesiology score; FNB, femoral nerve block; LFCNB, lateral femoral cutaneous nerve block

Postoperative complication rates also did not differ between the two groups (Table 2).

**Table 2:** Complications

	FNB + LFCNB group	FNB group	
PONV, n (%)	14 (33%)	15 (31%)	p=0.84 (95% CI -0.17 to 0.21)
Nerve injury, n	0	0	

CI, confidence interval; FNB, femoral nerve block; LFCNB, lateral femoral cutaneous nerve block; PONV, postoperative nausea and vomiting

**Figure 4:**
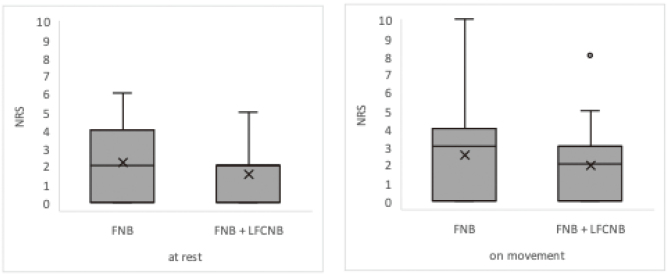
Pain at rest and during movement at 3 hours postoperatively The differences were not statistically significant. FNB, femoral nerve block; LFCNB, lateral femoral cutaneous nerve block

There was no significant difference in NRS scores at rest and during movement 3 h postoperatively (at rest: Z = -1.6814, p=0.0927; during movement: Z = -0.9677, p=0.9487) (Figure. 4).

**Figure 5:**
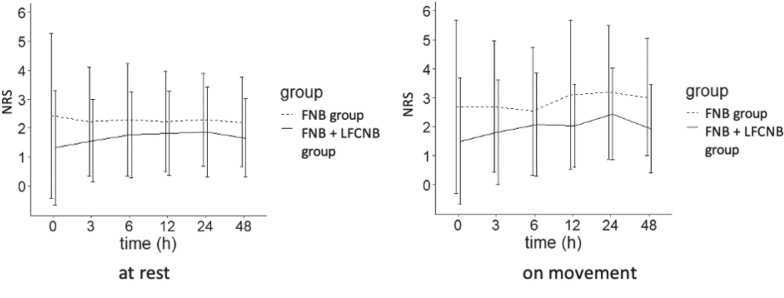
Pain at rest and during movement up to 48 hours postoperatively The differences between the two groups were not statistically significant. FNB, femoral nerve block; LFCNB, lateral femoral cutaneous nerve block

The change in NRS score up to 48 h postoperatively, a secondary endpoint, tended to be lower in the FNB + LFCNB group throughout the observation period, but there was no significant difference in the other measurement points between the two groups (Figure 5).

## Discussion

This study found no significant difference in NRS scores at rest after THA using DAA, regardless of LFCNB use. However, no significant difference was noted between the use and non-use of LFCNB. This result is unlikely to be accidental. It is reasonable to conclude that LFCNB did not yield a significant difference in NRS scores. For the secondary outcome, that is changes in NRS scores over time, NRS scores tended to be lower in the LFCNB group throughout the observation period. However, no significant differences were observed between the groups.

One possible reason for the lack of a significant difference between the FNB and FNB + LFCNB groups could be that the wound pain associated with THA using DAA was not as severe as that associated with other approaches. THA using DAA can preserve the muscles and nerves in the thigh^14)^. In fact, DAA has been shown to reduce postoperative pain compared with other approaches^4, 5, 15)^. In this study, a few patients did not require additional IV-PCA. Therefore, if femoral pain was relieved with FNB, pain at the incision site on the outer thigh was controlled with fentanyl via IV-PCA and was possibly not reflected in the postoperative NRS scores. No significant differences were observed between the FNB and FNB + LFCNB groups up to 48 hours postoperatively. Another possibility is that the administration of LFCNB after general anesthesia in this study might have caused the nerve block to fail. It is possible that some patients in the FNB + LFCNB group might have failed to receive a successful block. Otherwise, the reported success rate for ultrasound-guided LFCNB was 94.7–97.5%^16, 17)^. We set the dose to 10 mL in this study based on the report by Nielsen et al.^16)^, as it is known that a nerve block dose of ≤ 8 mL within the FFFT could reduce the success rate and extent of the drug effects^17, 18)^. Thus, the possibility of nerve block failure is low. It was difficult to confirm the clinical success of LFCNB and the extent of its effects due to the concomitant general anesthesia. Nevertheless, the blocks were performed by four experienced surgeons under ultrasound guidance, and there were no cases in which the LFCN could not be identified. The low possibility of inadequate extent of the drug effects was considered. Another possible cause for the lack of significant results is the anatomical diversity of the LFCN. Studies investigating the extent of pain relief in awake patients after LFCNB have revealed greater individual differences than previously thought^16, 18, 19)^. On the other hand, Nielsen et al.^16)^ reported that when a fascia iliaca compartment (FIC) block and an LFCNB were performed in the same patient, greater anesthetic coverage of the outer thigh was achieved with the FIC block, which was injected at a higher location than the LFCNB. This indicates that the use of the FIC block may be more desirable to ensure adequate anesthetic coverage of the outer thigh.

A limitation of this study is that the primary outcome was defined using the subjective NRS score, which by itself is not sufficient to accurately evaluate wound pain. The NRS scores increased when there was pain in other regions. Therefore, it is necessary to consider employing scales other than the NRS. For example, it is possible that the amount of additional analgesics (i.e., fentanyl) is more suitable. Another limitation is the decision regarding exactly when to evaluate the pain. We believe that the immediate postoperative period would be strongly influenced by intraoperative opioids which were used. Therefore, we decided to set the evaluation time at 3 h postoperatively, considering that the effects of the nerve block would persist, but the blood concentration of fentanyl were also likely to be stable by this time. Casati et al. reported that a preoperative nerve block using levobupivacaine provided analgesia up to 6 h postoperatively, and we believe that this endpoint time is reasonable^20)^. However, changes in the timings of the evaluation may have yielded different results.

In addition, the timing of the nerve block may also be a point of contention. Postoperative nerve blockade may have blocked acute pain and provided long-term pain relief, which again, may have shown different results.

In conclusion, LFCNB in patients undergoing THA using DAA did not demonstrate a significant difference in postoperative NRS scores when compared with IV-PCA of Fentanyl. In these cases, LFCNB did not improve postoperative pain relief.

## Funding

The authors did not receive funding or financial grant for this work.

## Author contributions

SS collected date and wrote the manuscript. TO helped edit the manuscript. SN performed statistical analysis. SS, KK, YS and MK helped performed nerve blocks. All authors read and approved the final manuscript.

## Conflicts of interest statement

The authors declare no competing or conflicts of interest in this work.
